# The Combined Effects of Additives on the Conventional and High-Temperature Performance Properties of Warm Mix Asphalt Binders

**DOI:** 10.3390/ma16247648

**Published:** 2023-12-14

**Authors:** Anna Chomicz-Kowalska, Joanna Bartos, Krzysztof Maciejewski, Mateusz M. Iwański

**Affiliations:** 1 Department of Transportation Engineering, Faculty of Civil Engineering and Architecture, Kielce University of Technology, 25-314 Kielce, Poland; jbartos@tu.kielce.pl (J.B.); kmaciejewski@tu.kielce.pl (K.M.); 2 Department of Building Engineering Technologies and Organization, Faculty of Civil Engineering and Architecture, Kielce University of Technology, 25-314 Kielce, Poland; matiwanski@tu.kielce.pl

**Keywords:** paving grade bitumen, polymer-modified bitumen, compaction aid, warm mix additive, high-temperature stiffness, non-recoverable creep compliance, MSCR, DSR

## Abstract

The present study investigates the effects of the simultaneous use of two additives, an organosilane warm mix asphalt (WMA) agent and a grade-bumping polyolefin compound, on the conventional and high-temperature performance properties of a paving grade 50/70 bitumen and a polymer-modified 45/80-55 bitumen. The WMA agent and polyolefin additive were introduced to the binders at rates of up to 0.3% and 2%, respectively. The base asphalt binders and their blends with the additives were tested before and after aging in a rolling thin film oven test at a temperature of 143 °C. The effects of the investigated additives were found to be dependent on the type of base binder and its aging state. It was generally observed that the WMA additive decreased the performance of the asphalt binders and limited the effects of the other additive, which increased the high-temperature stiffness and non-recoverable compliance of the blends. This interaction amounted to as much as an approx. 20% decrease in high-temperature stiffness and non-recoverable compliance of the binders. The additives caused a small increase in the elasticity of the binders and improved their creep performance when measured in multiple stress creep recovery tests.

## 1. Introduction

One of the means to decrease the energy intensity of the road construction industry is to utilize warm mix asphalt (WMA) techniques, which are typically characterized by processing temperatures between 20 °C and 30 °C lower than conventional asphalt paving processes [1].

To date, a number of field scale investigations confirmed that test sections produced with warm mix asphalt techniques were characterized by the decreased high-temperature performance of asphalt binders and asphalt mixtures [2,3,4]. These experiences show that, in many cases, the application of WMA techniques may hinder the early service performance of the asphalt mixtures in terms of their high-temperature performance. It was also shown that the high-temperature performance of the WMA mixtures improved significantly during the first two years of pavement service, to levels similar to those seen in conventionally produced pavements.

The lowered mixing temperatures of WMAs typically result in decreased aging of the asphalt binders. The aging of the asphalt binders during the production, transport and paving of asphalt mixtures is mainly caused by two processes: one being the oxidation reaction between the oxygen and the asphalt binder, while the second is identified as the volatilization of organic compounds [5,6]. Both of these processes are highly dependent on temperature [7]. Therefore, a number of studies have been conducted to evaluate the effects of aging temperature on the properties of WMA asphalt binders. The observations made in the field sections were confirmed in laboratory studies [8,9,10,11,12,13,14], showing a great dependence of the high-temperature properties of the asphalt binders on the laboratory aging protocols and the aging temperatures in particular. In studies comparing asphalt binders recovered from asphalt mixtures and the RTFOT-aged binders [15,16,17], it was shown that the standard temperature during short-term aging (163 °C) was too high to reflect the aging processes during the production of WMA mixtures, while 123 °C can be considered too low.

The methods for producing warm mix asphalt include binder foaming, the use of liquid and solid WMA additives [9,18,19,20,21,22,23], binder fluxing [24,25,26], direct water foaming [27,28,29,30], the introduction of asphalt mix additives [18,31,32] and combinations of some of the mentioned methods [33,34,35,36].

The application of certain processes and additives may, however, result in unexpected changes in the properties of the produced mixtures. Some popularly used anti-stripping agents and liquid warm mix additives may impact the dynamics of the aging processes in asphalt binders, decreasing the final asphalt binder stiffness resulting from technological and even long-term aging [7,33,37,38]. Such additives may also affect other processes during mix production, such as foaming [39]. The effects of these additives are, however, difficult to predict and specific to certain asphalt binders. Although the underlying mechanisms behind these effects are still not well understood, the postulated ones include the antioxidative and dispersive action of these additives. A different group of warm mix processes relies on the use of waxes of different origins, which not only decrease the processing temperatures of asphalt mixtures but often may enhance their high-temperature and moisture performance [40,41,42,43,44,45]. It was also found that some synthetic waxes have the capacity to slow down the formation of carbonyl compounds in asphalt binders and therefore decrease the aging-induced changes in their properties, as quantified, e.g., using aging indices [11,46,47].

Based on the state-of-the-art, it was concluded that an investigation into the means for improving the high-temperature performance of asphalt binders used in WMA mixtures would contribute to the subject area. To date, the most often studied group of liquid warm mix additives belongs to those based on fatty amine chemistry [48], whereas other types of formulations (e.g., organosilane compounds) are also available [49]. Therefore a silane-based liquid WMA additive was selected for this study, permitting significant reductions in processing temperatures and improvements in performance [50,51,52,53,54]. For improving the high-temperature performance of the binder, a polyethylene wax additive [55] was selected, due to its simple chemistry and the scarcity of studies into its application. The effects of the simultaneous use of these two additives were investigated with two bituminous binders—a paving grade bitumen and a polymer-modified bitumen. The paper focuses on the high-temperature performance properties of asphalt binders and on evaluating the interactions between the two incorporated additives.

## 2. Materials and Methods

### 2.1. Materials

#### 2.1.1. Asphalt Binders

To investigate the effects of the simultaneous use of the proposed additives, two distinctly different road paving bitumen were chosen as a baseline for the present study: a 50/70 paving grade bitumen and a 45/80-55 polymer-modified bitumen. Both asphalt binders are locally used, among other cases, typically for producing road wearing courses, and were obtained commercially from a local refinery (Orlen Asfalt, Płock, Poland). The basic characterization of these base asphalt binders is provided in Table 1.

#### 2.1.2. Asphalt Binder Additives

Two asphalt binder additives were used in the present study:

- A liquid WMA additive based on organosilane compounds used for decreasing production and paving temperatures of asphalt mixtures was designated as additive A [49];
- A solid pelletized additive (a polyethylene wax) used for the grade bumping of paving grade and polymer-modified asphalt binders and characterized particularly by a high compatibility with SBS-modified binders was designated as additive B [55].

**Figure 1:**
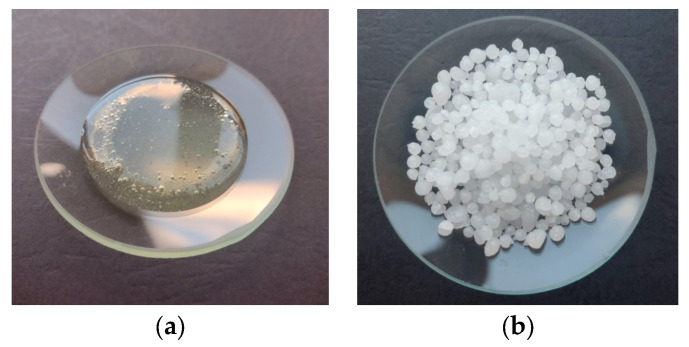
Photographs of the additives used in the study: additive A (**a**), additive B (**b**).

Properties of the additives are presented in Table 2 and their photographs can be found in Figure 1.

The dosing range of the WMA additive (A) was established to exceed the manufacturer’s recommended dosing range (to evaluate its possible negative effects) while the dosing of the solid additive (B) was set to be in the manufacturer’s recommended range.

The asphalt binders and the additives were blended for 15 min at 135 °C using a low-shear mixer.

### 2.2. Methods

#### 2.2.1. Testing Methods

The asphalt binders were tested for their basic classification properties (penetration, softening point and Fraass breaking point) using automated apparatuses. The assessment of performance properties was conducted using a direct shear rheometer (TA Instruments DHR-2, New Castle, DE, USA). The testing methodologies included:

- Conventional properties of non-aged binders:
- Penetration at 25 °C (EN 1426 [56]);
- Softening point (EN 1427 [57]);
- Fraass breaking point (EN 12593 [58]).
- Performance properties of non-aged and aged binders:
- High-temperature stiffness G*/sin(δ) based on the values of complex shear moduli (G*) and phase angle (δ) measured in oscillatory tests (AASHTO T315 [62], EN 14770 [63]);
- Multiple stress creep recovery (MSCR) tests for assessing non-recoverable creep compliance (J_nr 3.2 kPa_) and recovery (R_3.2 kPa_) at 3.2 kPa shear stress (AASHTO T350 [64], EN 16659 [65]).

The evaluated high-temperature stiffness was obtained at temperatures equal to the high-temperature grades of the base asphalt binders, that is 64 °C and 70 °C for the 50/70 and the 45/80-55 bitumen, respectively. The MSCR tests were performed for these binders at temperatures closer to real pavement temperatures, which were 58 °C and 64 °C, respectively. The different temperatures were utilized to obtain comparable responses in both groups of asphalt binders.

#### 2.2.2. Short-Term Aging of Asphalt Binders

The investigated asphalt binders were subjected to short-term laboratory aging conducted using the rolling thin film oven test (RTFOT) apparatus. The evaluated asphalt binder blends were tested as intended for use in WMA asphalt mixtures, therefore, a lower-than-typical RTFOT aging temperature was used of 143 °C, as it is regarded to be more representative to the processing of WMA mixtures [16,17]. The short-term aging procedure was based on the EN 12607–1 standard [66].

#### 2.2.3. Design of Experiment

A three-level, full factorial experimental design was employed to measure the combined effects of the organosilane and polyolefin additives on the properties of asphalt binders. The investigated dosing ranges of these additives included:

- Additive A: 0.00%, 0.15%, 0.30%;
- Additive B: 0.0%, 1.0%, 2.0%.

**Figure 2:**
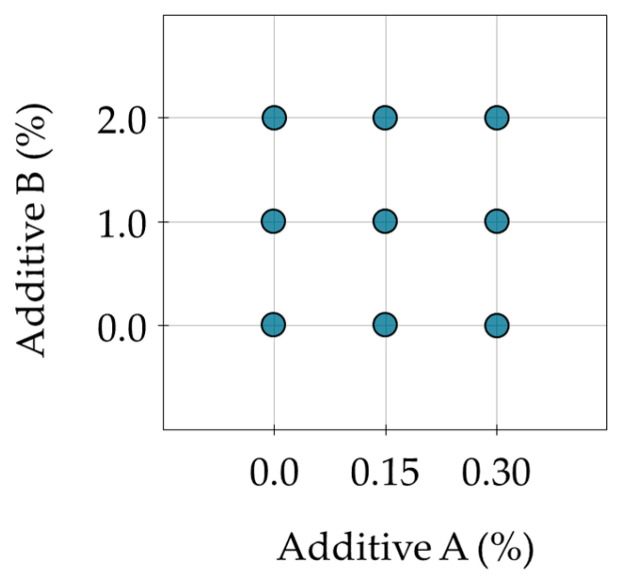
Three-level, full factorial experimental design implemented in the present study.

The implemented design for both asphalt binders, presented in Figure 2, enabled estimation of the linear, quadratic and interaction terms related to the effects of the investigated additives. In this design, the results of introducing the additives separately and in combination could be easily assessed. The experimental plan was repeated for both binders before and after the RTFOT aging for evaluation of the performance parameters. The number of replications in the experiments were as follows: penetration—6; softening point—6; Fraass breaking point—6; oscillatory DSR tests—3; and creep DSR tests—3. The reported results of the tests are reported in figures with the mean value and 95% confidence intervals (provided next to the data bars and in the form of error bars).

The effects associated with the additives introduced to the asphalt binders were quantified using linear statistical models with linear (L), quadratic (Q) and interaction terms as in Equation (1):

(1) $$Y=\beta_{0}+\beta_{1}X_{1}+\beta_{2}X_{2}+\beta_{3}X_{1}^{2}+\beta_{4}X_{2}^{2}+\beta_{5}X_{1}X_{2}$$

The effects and their significance were assessed using analysis of variance (ANOVA). As a result, the *p*-values based on the F statistic and the estimates of the model parameters were computed.

## 3. Results

### 3.1. Conventional Properties of Asphalt Binders

**Figure 3:**
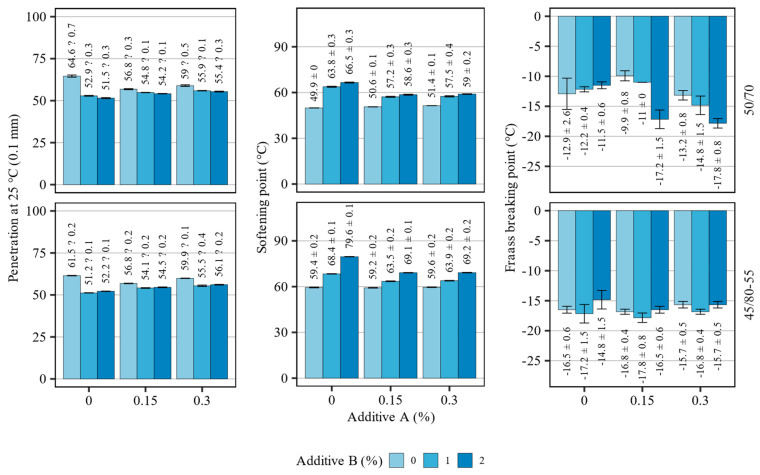
Effects of the investigated additives on the conventional properties of the investigated binders (means and 95% confidence intervals).

The results of the conventional tests for assessing penetration at 25 °C, softening point (ring and ball method) and Fraass breaking point are shown in Figure 3. The investigated binders, which were based on 50/70 and 45/80-55 bitumen, were tested directly after mixing with the additives, assuring adequate homogenization. The top and bottom rows of Figure 3 represent the blends based on the 50/70 and 45/80-55 binders, respectively.

The introduction of the additives had visible effects on both base asphalt binders. The major effects could be attributed to additive B, which caused significant changes in all evaluated parameters. The addition of the polyolefine compound in the amount of 1% caused a significant drop in the penetration of both binders. A further increase in its concentration did not result in visible changes in this scope. The most prominent changes were observed when additive A was not added which, at contents of 0.15% and 0.30%, decreased the effects of additive B.

In terms of the softening point, additive B caused an increase in the softening point of both binders, nearly proportional to its content. The greatest effects in this scope were observed, again, when additive A was not used. The 50/70 binder, modified solely with additive B, experienced the highest rate of increase in the softening point amounting to 13.9 °C at 1% of additive B. The use of additive B with the 45/80-55 binder resulted in the highest overall increase in this parameter that was seen at 2% of its concentration, which amounted to a 20.2 °C rise in the softening point. Additive A had minimal effects on the unmodified asphalt binders in this scope.

The additives had distinctly different effects on the Fraass breaking point values, depending on the type of the binder. In the case of the 45/80-55 base binder, the addition of modifiers had only minor effects in this scope, resulting in changes in the breaking point temperature amounting to typically less than 1 °C. On the other hand, the effects of the additives on the 50/70 asphalt binder were clearly pronounced, and their interaction has been observed. When additive A was not used, the addition of the polyolefin compound resulted in a small but consistent increase in the breaking point temperature. However, the addition of the organosilane agent inverted the effects of the polyolefin additive, causing it to decrease the breaking point. Finally, the lowest breaking point temperatures for the 50/70 asphalt binder were registered at 2% additive B when additive A was dosed at 0.15% and 0.30%.

Table 3 presents the results of the statistical evaluation of the effects of the additives on the conventional properties of both asphalt binders.

The analysis of variance of the presented results of penetration and softening point for both asphalt binders has shown that all of the evaluated effects (linear, quadratic and interactions) related to the introduced additives had a statistically significant impact on these parameters (df = 48, *p* < 0.05). In the evaluation of the Fraass breaking point, the same was true only for the 45/80-55 binders, whereas in the case of the 50/70 asphalt binders, only the quadratic and interaction terms were statistically significant (df = 48, *p* < 0.05). To summarize, both additives had a statistically significant impact on the values of all considered conventional properties of both asphalt binders.

### 3.2. Functional Properties of Asphalt Binders

The subsequent sections investigate the effects of the additives on the measured performance properties of the asphalt binders. Detailed summaries of the statistical analyses for assessing the effects of the additives on the measured responses are also provided in this section in the form of tables.

#### 3.2.1. High-Temperature Stiffness (G*/sin(δ))

**Figure 4:**
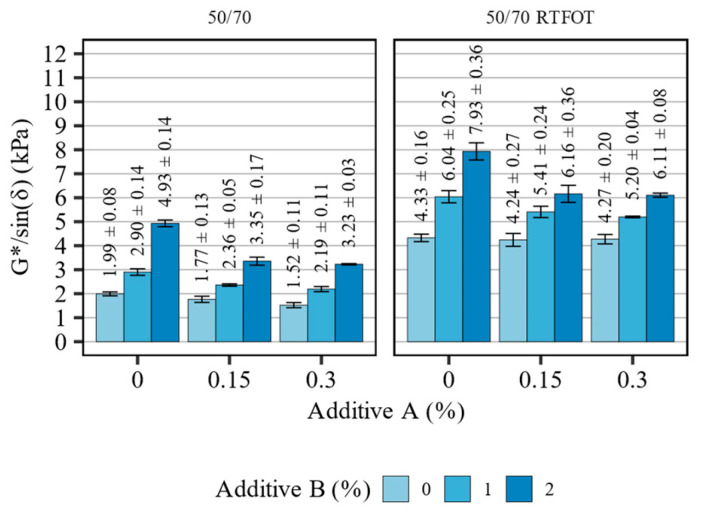
Results of G*/sin(δ) in terms of the effects of additives A and B on the 50/70 asphalt binders (means and 95% confidence intervals).

Figure 4 presents the high-temperature stiffness (G*/sin(δ)) results of the 50/70 asphalt binder blends before and after short-term aging, tested at the high-PG temperature of the base asphalt binder (64 °C). Table 4 summarizes the statistical analysis of the evaluated effects.

The results obtained in the oscillatory testing of the 50/70 asphalt binders indicate clear effects of both additives on their high-temperature stiffness, as well as an additional effect of the short-term aging to which they were subjected. Similarly to the softening point results, the most significant effects could be attributed to additive B, which caused significant increases in the stiffness of the non-aged and short-term aged binder blends. The addition of the organosilane agent alone has only resulted in the decrease in the high-temperature stiffness of the material, but only before the RTFOT aging. However, when the additives were used in combination, additive A significantly decreased the G*/sin(δ) values of the blends, regardless of aging. This observation was confirmed using statistical analysis, yielding relatively high interaction parameter estimates (−2.06 and −2.95) and small *p*-values (*p* < 0.001).

**Figure 5:**
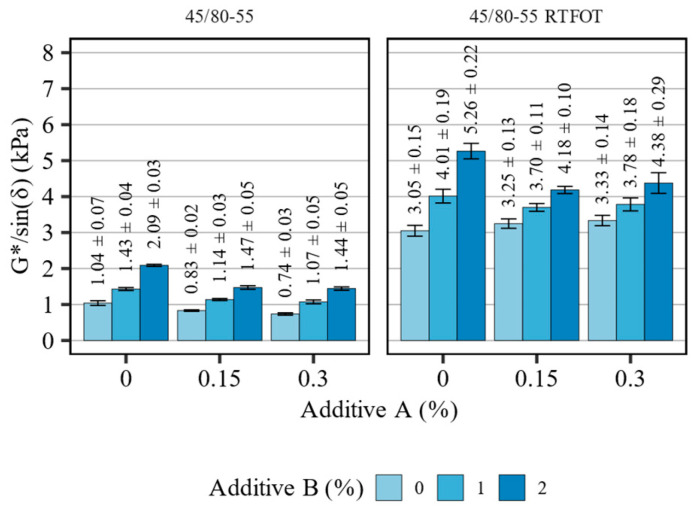
Results of G*/sin(δ) in terms of the effects of additives A and B on the 45/80-55 asphalt binders (means and 95% confidence intervals).

Figure 5 presents the high-temperature stiffness (G*/sinδ) measurement results of the 45/80-55 asphalt binder blends before and after short-term aging, tested at the high-PG temperature of the base asphalt binder (70 °C). Table 5 summarizes the statistical analysis of the evaluated effects.

The effects of the investigated additives on the 45/80-55 polymer-modified bitumen base were similar to those seen with the 50/70 paving grade binder, however, the measured effects were smaller in magnitude. In the non-aged asphalt binders, the organosilane additive consistently decreased the high-temperature stiffness of the binders, although the effect was observed to be strongest when the lesser (0.15%) dose of the modifier was introduced. On the other hand, the addition of this WMA agent without introducing the other additive, caused small increases in the G*/sin(δ) after the RTFOT aging. The addition of the polyolefin compound (additive B) again resulted in nearly proportional increasing in the high-temperature stiffness of the blends, regardless of the aging. This linear relationship can be inferred through the very small estimates of the quadratic terms in the models (0.05 and 0.08). These changes in high-temperature stiffness were, however, smaller than in the case of the 50/70 base binder.

**Figure 6:**
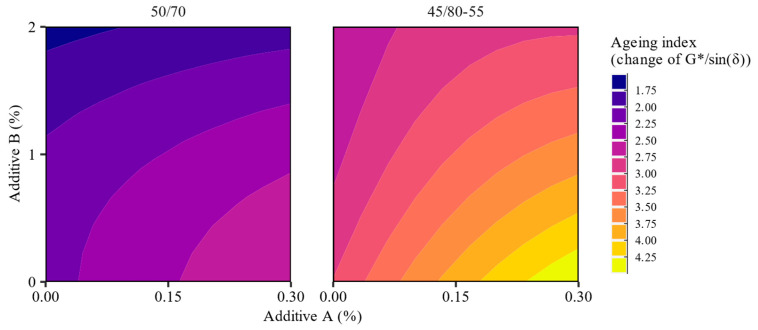
Effects of the investigated additives on the aging index of the respective asphalt binder blends.

Additional effects of the investigated additives could be observed in terms of the relative changes in the binders’ high-temperature stiffness after the RTFOT short-term aging, quantified using the aging index in Figure 6. The plots of the aging index (*AI*) represent ratios between the high-temperature stiffness of respective RTFOT aged and non-aged asphalt binder blends as in Equation (2):

(2) $$AI=\frac{G_{RTFOT}^{*}/\sin\left(\delta_{RTFOT}\right)}{G^{*}/\sin\left(\delta \right)}$$

The direct comparison of the high-temperature stiffness of the investigated asphalt binders before and after short-term aging using the aging index has shown that the evaluated changes in the G*/sin(δ) parameter were strongly influenced by both additives. The magnitude of these effects was larger in the polymer-modified bitumen, and in the case of both asphalt binders, additive A increased the aging indices, while the introduction of additive B contributed to their decrease. By inspection of Figure 4 and Figure 5, it can be stated that the high values of the aging indices in the 50/70 binders modified solely by the organosilane agent were mostly caused by the significant decrease in their high-temperature stiffnesses before aging. In other cases, these changes were more complex, where the effects seen both before and after the RTFOT contributed significantly. Additionally, when the investigated additives were simultaneously introduced at rates A: <0.15% and B: >1%, the aging indices of the resulting binder blends were usually smaller than those of the base asphalt binders.

#### 3.2.2. Multiple Stress Creep Recovery Performance

**Figure 7:**
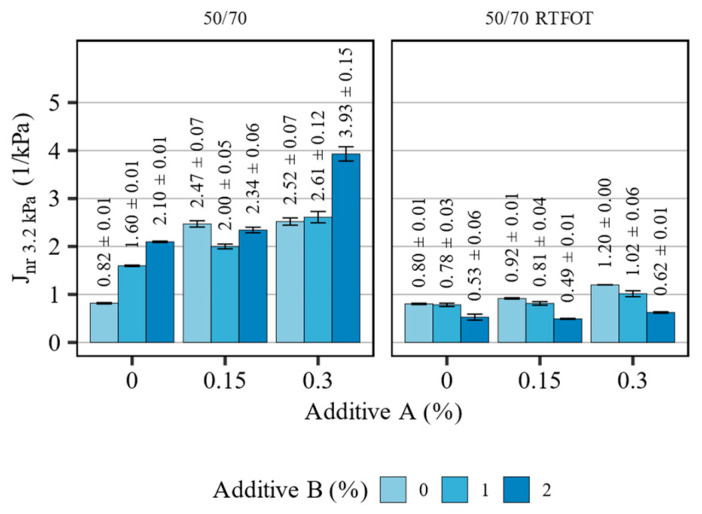
Results of J_nr 3.2kPa_ in terms of the effects of additives A and B on the 50/70 asphalt binders (means and 95% confidence intervals).

Figure 7 presents the measurement results of the non-recoverable creep compliance (J_nr 3.2kPa_) of the 50/70 asphalt binder blends before and after short-term aging, tested at 58 °C. Table 6 summarizes the statistical analysis of the evaluated effects.

The investigated additives affected the non-recoverable creep compliance of the 50/70 asphalt binder blends significantly and these effects were different depending on the aging state of the binders. The introduction of both additives to the non-aged binder resulted in major increases in the J_nr 3.2kPa_ parameter. Most prominently, the addition of the polyolefin compound increased the non-recoverable compliance of the non-aged binder, which was not in line with the findings from the oscillatory measurements. The effects of additive B in this scope were also highly inconsistent, which can be seen in the figure and in high recorded *p*-values (*p* > 0.05). The addition of the organosilane agent magnified this effect further (estimate of linear effect: 4.963, *p*-value = 0.033), doubling the J_nr 3.2kPa_ values when 0.3% of additive A and 2% of additive B were used. These effects might contribute to decreasing the compaction effort in asphalt mixtures with these additives. The short-term aging has significantly changed the effects of the polyolefin compound, causing it to significantly decrease the non-recoverable compliance of all asphalt binder blends. The organosilane additive still increased the J_nr 3.2kPa_ values, however, this effect was significantly smaller compared to the non-aged binders.

**Figure 8:**
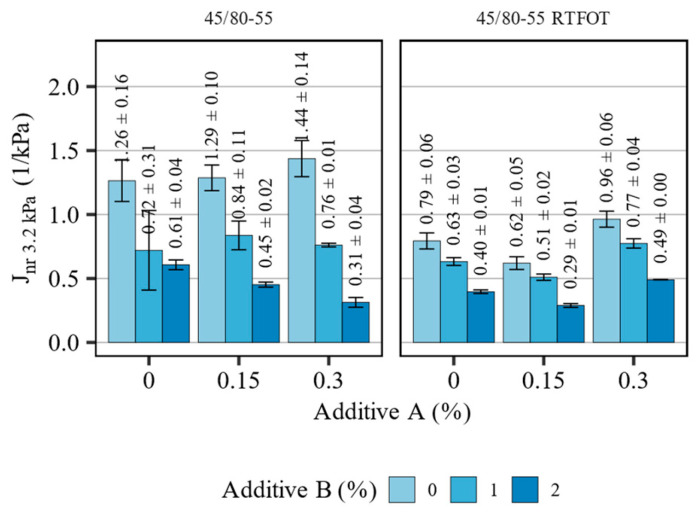
Results of J_nr 3.2kPa_ in terms of the effects of additives A and B on the 45/80-55 asphalt binders (means and 95% confidence intervals).

Figure 8 presents the measurement results of the non-recoverable creep compliance (J_nr 3.2kPa_) of the 45/80-55 asphalt binder blends before and after short-term aging, tested at 64 °C. Table 7 summarizes the statistical analysis of the evaluated effects.

Changes in the non-recoverable creep compliance due to the introduction of the additives and aging were more predictable in the case of the polymer-modified asphalt binder. The obtained results were mostly in line with the characteristics obtained in the oscillatory measurements and softening point tests. In the non-aged binder blends, additive A on its own only slightly increased the J_nr 3.2kPa_ values (significance of the linear effect: *p* = 0.056), significance of quadratic effect: *p* = 0.765)_,_ whereas the introduction of the polyolefin compound has revealed a significant interaction between the additives (effect estimate −0.78, *p* < 0.001), dependent on their concentration. Most prominently, when the 2% concentration of additive B was used, the organosilane agent magnified its effects in decreasing the non-recoverable compliance. Before aging, the contribution of additive A was observed in interaction with the other additive (*p* < 0.001). However, after the RTFOT aging, additive A had a minor role in shaping this creep characteristic. The introduction of additive B, the polyolefin compound, had the potential to halve the values of the non-recoverable creep compliance of the 45/80-55 asphalt binder, particularly when additive A was not used or added at a dose of 0.15%.

**Figure 9:**
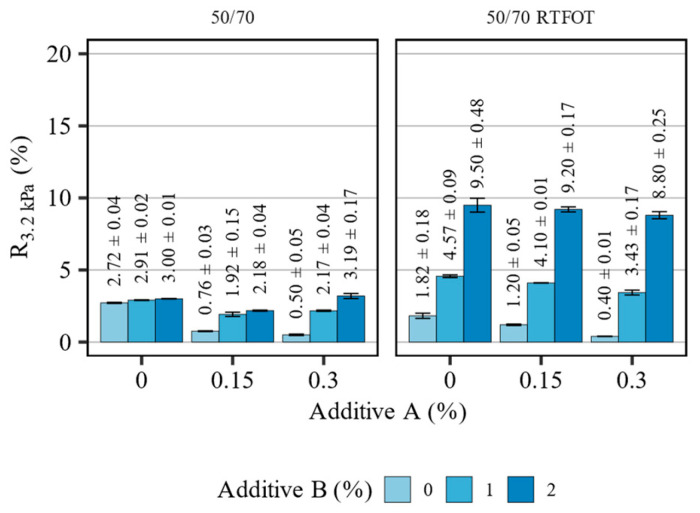
Results of R3.2 kPa in terms of the effects of additives A and B on the 50/70 asphalt binders (means and 95% confidence intervals).

Figure 9 presents the measurement results of recovery (R_3.2kPa_) in the MSCR tests of the 50/70 asphalt binder blends before and after short-term aging, tested at 58 °C. Table 8 summarizes the statistical analysis of the evaluated effects.

The asphalt binder blends based on the 50/70 bitumen exhibited low values of recovery in the MSCR tests, typical for paving grade asphalt binders lacking elastomeric modification. In the non-aged condition, the measured values of R_3.2kPa_ peaked at approx. 3%. The introduction of additive A resulted in a decrease in the recovery values from 2.72% of the base binder to 0.76% and 0.50% at 0.15% and 0.30% concentrations. The introduction of additive B restored the recovery values to the proximity of base levels. After the RTFOT aging, the observed effects were similar, but their magnitude was greatly increased, particularly in the case of the polyolefin compound. The introduction of additive B caused a significant increase in the measured recovery, up to the range of 8.8–9.5% when 2% of this additive was used. Given that additive B is not an elastomer, these recovery values were not expected to exceed 30%, as the base asphalt binder did not exhibit a significant delayed elastomeric response. Both evaluated additives contributed significantly (*p* < 0.05) to the measured responses, however, the magnitude of the recovery characteristics deem them not significant in engineering considerations. The effects of the additives on the recovery of the 50/70 asphalt binder can be regarded as nonconsequential due to the overall low level of this response.

**Figure 10:**
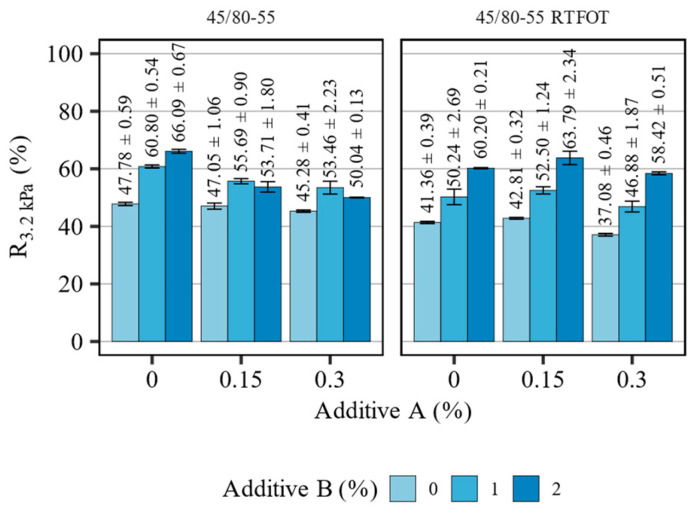
Results of R_3.2kPa_ in terms of the effects of additives A and B on the 45/80-55 asphalt binders (means and 95% confidence intervals).

Figure 10 presents the measurement results of recovery (R_3.2kPa_) in the MSCR tests of the 45/80-55 asphalt binder blends before and after short-term aging, tested at 64 °C. Table 9 summarizes the statistical analysis of the evaluated effects.

The 45/80-55 polymer-modified bitumen exhibited high values of recovery, both before and after the RTFOT short-term aging. The introduced additives significantly, and in different ways, affected the elastomeric response of the investigated asphalt binder blends. When the polyolefin compound was introduced to the non-aged binder solitarily, the measured recovery increased from 47.8% to 60.8% and 66.1% at 1% and 2% additive content, respectively. The addition of the WMA agent decreased the effects of additive B significantly, and this action coincided with the concentration of additive A. This observation is validated by the high value of the A:B interaction term in the evaluated statistical model (effect estimate: −22.57, *p* < 0.001). In the RTFOT-aged asphalt binders, the WMA additive had only a minor influence on the recorded values of recovery, which were found to increase slightly at 0.15% concentration of the additive and decrease by no more than approx. four percentage points at 0.30% concentration. After short-term aging, the base binder experienced an approx. six percentage point drop in the recovery, but the addition of the polyolefin compound increased its values despite the use of additive A. The statistical analysis (Table 9) has shown that both additives significantly affected the measured variable. Similarly, as it was before the RTFOT, additive B had a greater impact on recovery than the WMA agent. In this instance, however, the effects of the interaction between the additives were smaller, although still statistically significant (effect estimate: 4.17, *p* = 0.001).

**Figure 11:**
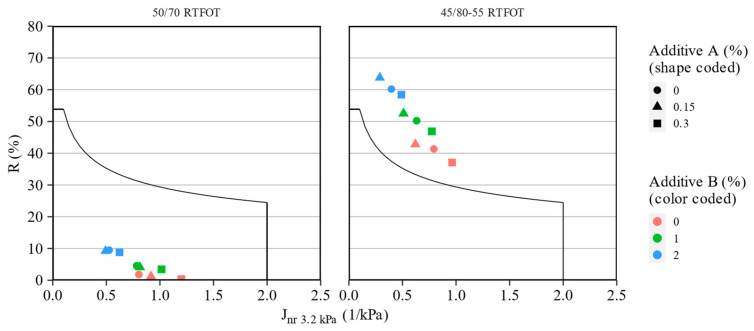
Relationships between non-recoverable creep compliance and recovery in the MSCR tests for assessing the elasticity of the investigated asphalt binders.

Figure 11 presents the MSCR test results of the 50/70 and 45/80-55 asphalt binder blends after short-term aging, tested at 58 °C and 64 °C, respectively. The relationships between the recovery (R_3.2kPa_) and non-recoverable creep compliance (J_nr 3.2kPa_) obtained in the tests are presented for all investigated asphalt binder blends. The amounts of the additives are presented by the shape (additive A) and color (additive B) of the data points. The curve presented in the figure follows Equation (3) introduced to the AASHTO M322 standard as a means for assessing the elasticity of the asphalt binders containing elastomeric polymers:

(3) $$y=29.37x^{-0.2633}$$

Figure 11 shows that the 50/70 paving grade bitumen blends do not meet the specification for an adequate elastomeric response. Despite this, additive B increased slightly the recovery values of the 50/70 asphalt binder, as shown earlier. Additive A had negligible effects in this scope. On the other hand, the effects of the investigated additives on the polymer-modified binder were significant. The introduction of the WMA agent increased the elastic response and decreased the non-recoverable creep compliance of the 45/80-55 asphalt binder, with the most favorable effects being recorded at the 0.15% concentration of the additive. The polyolefin compound had even greater effects, specifically on the recovery of the tested binders, despite the lack of mentioned elastomeric properties of this additive. It can be stated that the introduction of these additives improved both the creep performance and the elasticity of the polymer-modified bitumen.

## 4. Discussion

In the evaluation of the conventional properties of the asphalt binders, it was found that the WMA agent had only small effects on their penetration and softening points. On the other hand, the polyolefin compound had major effects on these characteristics of both asphalt binders, decreasing the penetration by approx. 10 units (0.1 mm) and increasing the softening point by up to 20 °C. The effects of these additives on the Fraass breaking point were quite significant in the 50/70 paving grade bitumen and pointed to the presence of a strong interaction between additives A and B. The simultaneous use of these additives decreased the measured breaking point from approx. −13 °C to approx. −18 °C. Although typically the low-temperature performance can be, to some degree, inferred based on the Fraass breaking point [67], the low-temperature performance characteristics of such blends should be investigated in the scope of binder aging, as further testing showed the strong dependence of other investigated binder parameters on the aging state.

The detailed analysis of the high-temperature functional properties of the asphalt binder blends revealed, in most cases, strong interactions between the investigated additives. In general, the WMA additive impaired, to some degree, the high-temperature characteristics of the tested binder blends, while the polyolefin compound considerably increased the high-temperature stiffness and decreased the non-recoverable creep compliance. The interaction effects were, in most cases, responsible for reducing the favorable effects of the polyolefin additive, which amounted to 18–22% changes in the high-temperature stiffness and non-recoverable compliance of the full A and B additive bitumen blends compared to the blend only containing additive B. In most cases, the effects of the investigated additives were dependent on the aging state of the tested binders. The RTFOT aging, in some cases, had profound effects on the measured responses, e.g., strongly magnifying their effects (e.g., G*/sin(δ) in both binders) or even inverting the relationships found in the non-aged binders (e.g., J_nr 3.2kPa_ in 50/70 binder, recovery in 45/80-55 binder).

As shown in Figure 6, the addition of the polyolefin additive significantly reduced the magnitude of the changes in stiffness caused by short-term aging. This may potentially result in the favorable long-term performance of the binders with this compound added. Additionally, as deduced from the data presented in Figure 11, none of the additives had a negative impact on the elasticity of the asphalt binders, which was a significant observation, specifically regarding the polymer-modified bitumen. In a number of studies, it was shown that the preservation of this characteristic is key for the adequate performance of highly stressed pavements [68,69,70].

Further work in this area could be devoted to evaluating the effects of ultraviolet aging [71,72,73] on the properties of asphalt binders with different additives used in WMA techniques, given it is a different and substantial mode of aging and a significant gap in this area persists [5].

## 5. Conclusions

Based on the premise that warm mix asphalt processes may result in the decreased high-temperature performance of asphalt mixtures, an investigation of asphalt binders intended for WMA mixtures was carried out. The present study investigated the effects of simultaneously incorporating two additives into paving grade and polymer-modified asphalt binders. The study included a liquid organosilane WMA additive utilized for decreasing the processing temperatures and a polyolefin compaction aid/grade-bumping additive for enhancing the high-temperature performance of the asphalt binder.

The major findings of the study can be summarized as follows:

- The effects of the additives introduced separately to the asphalt binders were in line with those found with other, similar products. The WMA additive alone had only small effects on the high-temperature properties of the asphalt binders, while the grade-bumping additive significantly improved their high-temperature performance;
- Significant interactions of the two additives, when used simultaneously, were discovered, resulting in a decreased efficacy of the grade-bumping additive, specifically when the dosing of additive A exceeded the recommended values;
- Short-term RTFOT aging had a major impact on the effects of the investigated additives.

Based on the findings shown in the study, it is recommended to thoroughly investigate the above-mentioned effects when different asphalt binder additives are used simultaneously.

Further studies in this area should be conducted, given that the simultaneous utilization of these types of additives may result in favorable changes in the characteristics of WMA asphalt binders. Despite the favorable changes in the high-temperature performance of the binders, the effects of these additives used together on other performance characteristics remain unknown. Particularly, the effects on fatigue and low-temperature performance should be investigated. Additional work should be directed to evaluating the effects of ultraviolet aging mechanisms in WMA surface course materials.

## Figures and Tables

**Table 1:** Properties of the asphalt binders used in the study.

Property	Unit of Measurement	Base Bitumen		Testing Method
		50/70	45/80-55	
Penetration at 25 °C	0.1 mm	64.6	61.5	EN 1426 [56]
Softening point	°C	49.9	59.4	EN 1427 [57]
Fraass breaking point	°C	−12.9	−16.5	EN 12593 [58]
Penetration index	-	−0.61	1.56	EN 12591 [59]
Dynamic viscosity at 135 °C	Pa·s	0.42	1.06	EN 13302 [60]
Performance grade	-	64−22	70−22	AASHTO M320 [61]

**Table 2:** Properties of the additives used in the study.

Property	Additive A	Additive B
Form	Viscous liquid	Solid pellets, 2–3 mm
Color	Yellow	White
Density (g/cm^3^)	1.01	0.93
Viscosity at 20 °C (mPa∙s)	120	-
Typical dosing range (by wt. of asphalt binder)	0.05–0.15% [49]	0.5–3.0% [55]
Role	WMA additive	Grade bumping additive

**Table 3:** Summary of analysis of variance (*p*-values) in terms of the effects of additives A and B on the conventional properties of 50/70 and 45/80-55 asphalt binders.

*p*-Values Effect:	Model Term		50/70			45/80-55		
		df	Penetration at 25 °C	Softening Point	Fraass Breaking Point	Penetration at 25 °C	Softening Point	Fraass Breaking Point
Intercept	$\beta_{0}$	1	<0.001	<0.001	<0.001	<0.001	<0.001	<0.001
Additive A (L)	$\beta_{1}$	1	<0.001	<0.001	0.063	0.012	<0.001	0.009
Additive B (L)	$\beta_{2}$	1	<0.001	<0.001	0.081	<0.001	<0.001	0.001
Additive A (Q)	$\beta_{3}$	1	0.007	<0.001	0.040	0.016	<0.001	<0.001
Additive B (Q)	$\beta_{4}$	1	<0.001	<0.001	0.033	<0.001	0.013	<0.001
A:B (L) interaction	$\beta_{5}$	1	<0.001	<0.001	<0.001	<0.001	<0.001	0.017
Residuals		48						
Adj. R^2^			0.820	0.956	0.629	0.848	0.973	0.494
Model *p*-value			<0.001	<0.001	<0.001	<0.001	<0.001	<0.001

**Table 4:** Summary of analysis of variance for the results of G*/sin(δ) in terms of the effects of additives A and B on the 50/70 asphalt binders.

G*/sin(δ) Effect:	Model Term		50/70			50/70 RTFOT		
		df	Estimate	Std. Error	*p*-Value	Estimate	Std. Error	*p*-Value
Intercept	$\beta_{0}$	1	2.03	0.098	<0.001	4.42	0.107	<0.001
Additive A (L)	$\beta_{1}$	1	−5.15	1.134	<0.001	−5.06	1.239	0.001
Additive B (L)	$\beta_{2}$	1	0.72	0.170	<0.001	1.75	0.185	<0.001
Additive A (Q)	$\beta_{3}$	1	13.35	3.440	0.001	16.60	3.758	<0.001
Additive B (Q)	$\beta_{4}$	1	0.31	0.077	0.001	−0.04	0.084	0.618
A:B (L) interaction	$\beta_{5}$	1	−2.06	0.364	<0.001	−2.95	0.398	<0.001
Residuals		21		0.189			0.207	
Adj. R^2^					0.964			0.968
Model *p*-value					<0.001			<0.001

**Table 5:** Summary of analysis of variance for the results of G*/sin(δ) in terms of the effects of additives A and B on the 45/80-55 asphalt binders.

G*/sin(δ) Effect:	Model Term		45/80-55			45/80-55 RTFOT		
		df	Estimate	Std. Error	*p*-Value	Estimate	Std. Error	*p*-Value
Intercept	$\beta_{0}$	1	1.05	0.031	<0.001	3.14	0.086	<0.001
Additive A (L)	$\beta_{1}$	1	−2.94	0.368	<0.001	−2.43	1.001	0.024
Additive B (L)	$\beta_{2}$	1	0.38	0.055	<0.001	0.83	0.150	<0.001
Additive A (Q)	$\beta_{3}$	1	6.86	1.116	<0.001	11.51	3.035	0.001
Additive B (Q)	$\beta_{4}$	1	0.05	0.025	0.042	0.08	0.068	0.265
A:B (L) interaction	$\beta_{5}$	1	−0.57	0.118	<0.001	−1.95	0.321	<0.001
Residuals		21		0.062				0.167
Adj. R^2^				0.975				0.935
Model *p*-value				<0.001				<0.001

**Table 6:** Summary of analysis of variance for the results of J_nr 3.2kPa_ in terms of the effects of additives A and B on the 50/70 asphalt binders.

J_nr 3.2kPa_ Effect:	Model Term		50/70			50/70 RTFOT		
		df	Estimate	Std. Error	*p*-Value	Estimate	Std. Error	*p*-Value
Intercept	$\beta_{0}$	1	1.21	0.189	<0.001	0.81	0.006	<0.001
Additive A (L)	$\beta_{1}$	1	4.96	2.181	0.033	0.18	0.080	0.038
Additive B (L)	$\beta_{2}$	1	−0.19	0.327	0.563	0.08	0.012	<0.001
Additive A (Q)	$\beta_{3}$	1	−0.41	6.613	0.952	3.76	0.243	<0.001
Additive B (Q)	$\beta_{4}$	1	0.29	0.149	0.062	−0.11	0.005	<0.001
A:B (L) interaction	$\beta_{5}$	1	0.22	0.701	0.760	−0.50	0.025	<0.001
Residuals		21		0.364			0.013	
Adj. R^2^					0.796			0.996
Model *p*-value					<0.001			<0.001

**Table 7:** Summary of analysis of variance for the results of J_nr 3.2kPa_ in terms of the effects of additives A and B on the 45/80-55 asphalt binders.

J_nr 3.2kPa_ Effect:	Model Term		45/80-55			45/80-55 RTFOT		
		df	Estimate	Std. Error	*p*-Value	Estimate	Std. Error	*p*-Value
Intercept	$\beta_{0}$	1	1.22	0.034	<0.001	0.77	0.014	<0.001
Additive A (L)	$\beta_{1}$	1	0.80	0.394	0.056	−2.12	0.162	<0.001
Additive B (L)	$\beta_{2}$	1	−0.56	0.059	<0.001	−0.09	0.024	0.002
Additive A (Q)	$\beta_{3}$	1	−0.36	1.196	0.765	8.98	0.492	<0.001
Additive B (Q)	$\beta_{4}$	1	0.12	0.026	<0.001	−0.05	0.011	<0.001
A:B (L) interaction	$\beta_{5}$	1	−0.78	0.126	<0.001	−0.13	0.052	<0.001
Residuals		21		0.065			0.027	
Adj. R^2^					0.970			0.982
Model *p*-value					< 0.001			< 0.001

**Table 8:** Summary of analysis of variance for the results of R_3.2kPa_ in terms of the effects of additives A and B on the 50/70 asphalt binders.

R_3.2kPa_ Effect:	Model Term		50/70			50/70 RTFOT		
		df	Estimate	Std. Error	*p*-Value	Estimate	Std. Error	*p*-Value
Intercept	$\beta_{0}$	1	2.65	0.049	<0.001	1.83	0.041	<0.001
Additive A (L)	$\beta_{1}$	1	−17.69	0.576	<0.001	−3.72	0.483	<0.001
Additive B (L)	$\beta_{2}$	1	0.69	0.086	<0.001	1.60	0.072	<0.001
Additive A (Q)	$\beta_{3}$	1	35.37	1.748	<0.001	−3.69	1.467	0.020
Additive B (Q)	$\beta_{4}$	1	−0.28	0.039	<0.001	1.12	0.033	<0.001
A:B (L) interaction	$\beta_{5}$	1	4.01	0.185	<0.001	1.21	0.155	<0.001
Residuals		21		0.096			0.080	
Adj. R^2^					0.989			0.999
Model *p*-value					<0.001			<0.001

**Table 9:** Summary of analysis of variance for the results of R_3.2kPa_ in terms of the effects of additives A and B on the 45/80-55 asphalt binders.

R_3.2kPa_ Effect:	Model Term		45/80-55			45/80-55 RTFOT		
		df	Estimate	Std. Error	*p*-Value	Estimate	Std. Error	*p*-Value
Intercept	$\beta_{0}$	1	48.22	0.634	<0.001	41.28	0.297	<0.001
Additive A (L)	$\beta_{1}$	1	−29.61	7.325	0.001	38.74	3.430	<0.001
Additive B (L)	$\beta_{2}$	1	18.33	1.098	<0.001	8.09	0.514	<0.001
Additive A (Q)	$\beta_{3}$	1	78.08	22.213	0.002	−177.95	10.401	<0.001
Additive B (Q)	$\beta_{4}$	1	−4.99	0.499	<0.001	0.74	0.234	0.005
A:B (L) interaction	$\beta_{5}$	1	−22.57	2.356	<0.001	4.17	1.103	0.001
Residuals		21		1.224			0.573	
Adj. R^2^					0.965			0.996
Model *p*-value					<0.001			<0.001

## Data Availability

Data available on request.
